# Metagenomics Reveal Correlations Between Microbial Organisms in Soils and the Health of *Populus euphratica*

**DOI:** 10.3389/fmicb.2020.02095

**Published:** 2020-09-08

**Authors:** Yu Tuo, Zhibao Dong, Xiping Wang, Beibei Gao, Chunming Zhu, Fei Tuo

**Affiliations:** ^1^School of Geography and Tourism, Shaanxi Normal University, Xi’an, China; ^2^College of Horticulture, Northwest A&F University, Yangling, China; ^3^Department of Pesticide Science, College of Plant Protection, Nanjing Agricultural University, State and Local Joint Engineering Research Center of Green Pesticide Invention and Application, Nanjing, China; ^4^Greening Committee Office of Forestry Bureau of Yulin City, Yulin, China

**Keywords:** *Populus euphratica*, Mu Us Desert, biological diversity, microbial communities, pathogen

## Abstract

Biological diversity plays an important role in the stability of ecosystems. The Mu Us Desert (MUD), located in Northern China, is an aeolian desert. Although it has been governed by a series of ecological restoration programs, the MUD still has limited biological diversity. *Populus euphratica* (*P. euphratica*), a xerophytic plant, has great potential to improve the biological diversity of the MUD. However, the survival rate of *P. euphratica* in the MUD has been very low. The current study tried to explore the mechanism of the high death rate of *P. euphratica* in the microbiome perspective. The correlation study between soil community composition and soil properties showed that water-filled pore space (WFPS), pH, EC, AP, NO_3_^–^, and NH_4_^+^ possess higher potential to change the bacterial community (18%) than the fungal community (9%). Principal coordinate analysis indicated that the composition of both bacteria (*Proteobacteria* and *Bacteroidetes*) and fungi (*Ascomycota*) in the root soil can be increased by *P. euphratica*. By systematically comparing between the fungal diversity in the root soil around *P. euphratica* and the pathogenic fungus extract from the pathogenic site of *P. euphratica*, we found that the high death rate of *P. euphratica* was associated with specific pathogenic fungus *Alternaria alternate* and *Didymella glomerata*. In addition, the microbiome composition analysis indicated that *P. euphratica* planting could also influence the portions of bacteria community, which also has great potential to lead to future infection. However, as the extraction and separation of bacteria from plants is challenging, the correlation between pathogenic bacteria and the high death rate of *P. euphratica* was not studied here and could be explored in future work.

## Highlights

- Soil microbiome can be influenced significantly by *Populus euphratica*.
- *A. alternate* and *D. glomerata* in soil can lead to the high death rate of *Populus euphratica*.
- Plant-pathogen interactions within bacteria and *Populus euphratica* death was observed.

## Introduction

The Mu Us Desert (MUD) was once covered by 81 km^2^ of sand dunes. Fortunately, after the efforts of several generations, its ecological environment has been greatly improved. Since 1978, the desert region in the MUD has been moved 400 km to the north (Feng et al., 2013; Li et al., 2013; Lai et al., 2016). The sand retreat was a great achievement and victory that made the MUD one of the most famous regional vegetation restorations (Li et al., 2019; Sun et al., 2019). However, the achievement of environmental management in the MUD is mainly attributed to the introduction of two kinds of plants: conifer pine and shrub. It is difficult for other conventional green plants to survive in the MUD due to the natural climate, and as a result, most of the restored areas were dominated by these two kinds of plants. Although proliferation of these species temporarily controlled the spread of the local desert and improved the local vegetation coverage, the limited biodiversity in the region renders the ecological environment fragile. The resulting risk of secondary desertification has always been an urgent problem for the ecological stability of the MUD region (Shu et al., 2018; Li et al., 2019).

YuLin (Yuyang District), located in the interior of the MUD, possesses 118.27 km^2^ of grassland reclamation. YuLin is one of most typical vegetated areas of the MUD and has great potential as a pilot site due to its ecological stability (Li et al., 2019). *Populus euphratica*, a perennial woody plant with high salinity and aridity tolerance, is widely spread in Western China and adjacent Central Asian countries (Keram et al., 2019). Considering the growth habit of *P. euphratica*, the climatic and environmental conditions of the Yuyang District can be suitable for its growth (Shu et al., 2018). However, after several years of trying, the success of *P. euphratica*, which should be able to improve the ecological development in the MUD, has stagnated due to its high mortality rate. At the same time, some factors governing the success of *P. euphratica* have been discovered. After these *P. euphratica* trees were planted in the nursery garden, the survival rate was close to 100% within the first year. Once the *P. euphratica* trees were transferred outside, almost 50% of the saplings died within the second year. During the third year, less than 5% of these *P. euphratica* trees survived. Only 5% of these tested trees grew healthy after a probationary period of 3 years. By analyzing the dead plants, some typical symptoms, like stem cankers and rust disease, etc., were found. The symptoms identified in almost all of the dead *P. euphratica* were consistent. These fatal diseases seem to be a reliable reason for the high death rate of *P. euphratica*. At the same time, why was there such a large difference in the death rate among different growth periods? Where or how were the *P. euphratica* trees infected?

Previous studies indicated that, the plant-associated microbial communities could be affected by the tissue age of the plant, environmental conditions, and agronomical practices (Vorholt, 2012; Leff et al., 2015; Arrigoni et al., 2018). In this plant–pathogen interaction system, microbiome plays a vital role for the health of the plant. The microbiome transplanted by the soil could always predetermine future plant health (Wei et al., 2019). This study is focused on the bacterial and fungal community in the soil around *P. euphratica* and the endophyte pathogenic to *P. euphratica*. The biological diversity in different places at different periods was studied by high-throughput sequencing. In addition, the endophytes pathogenic to *P. euphratica* were extracted, isolated, and authenticated by DNA sequencing. We analyzed the correlations between microorganisms in soils and endophytes in the pathogenetic *P. euphratica*.

## Materials and Methods

### Sampling Sites

According to the tree age, four different sampling sites were arranged: Sampling Site 1 (half year), the new development area of Yulin where *P. euphratica* had not been transplanted (XKWY, 38°9′37.7″N, 109°41′9.2″E) in the MUD, Yu Yang District, Northwest China; Sampling Site 2 (1 year), the new development area of Yunlin where *P. euphratica* were transplanted (XKY, 38°26′38.0″N, 109°35′38.2″E) in the MUD, Yu Yang District, Northwest China; Sampling Site 3 (2 years), the afforestation land (ZLD, 38°25′58.2″N, 109°37′18.7″E) in the MUD, Yu Yang District, Northwest China; Sampling Site 4 (3 years), Airport Road study site (JCL, 38°23′11.2″N, 109°37′11.78026″E) in the MUD, Yu Yang District, Northwest China. At the JCL study site, three types of different soils were investigated: JCLH, the soil around the surviving *P. euphratica* after 3 years of planting in JCL; JCLS, the soil around the dead *P. euphratica* after 3 years of planting in JCL; and JCLYLT, the soil in JCL without *P. euphratica* planting.

### Collection of Soil and Plant Samples

Six replications were conducted at all sampling sites of the selected soil samples. Soil samples were collected surrounding the soil stem, approximately 20 cm in diameter, of *P. euphratica* at depths from 10 to 50 cm. After thoroughly mixing, 100 g of prepared soil samples was stored at −80°C for sequencing. Corresponding air-dried soil samples were sieved by a 2-mm mesh and stored at 4°C for the analysis of soil physicochemical properties. The diseased parts of *P. euphratica* at different study sites were imaged and collected by cutting off the target stem.

### Isolation of Plant Endophytes

Twenty-four pathogenetic *P. euphratica* samples were collected from XKY, ZLD, and JCL. The collected samples were disinfected by 75% ethanol for 1 min and 10% NaClO for 5 min (surface treatment). After then, the disinfected samples were rinsed thoroughly by sterile water and then dried in aseptic fume hood. Finally, the sterilized symptomatic stems were cultured by potato dextrose agar (PDA) culture medium, with antibiotic supplements (50 μg/ml of ampicillin and streptomycin sulfate), at 25°C. The characteristics of cultured mycelium on PDA were observed daily and isolated. The microscopic morphological characteristics of isolated colony were observed and recorded (Olympus, Tokyo, Japan). All the valuable biological materials have been protected, controlled, and accounted according the laboratory biosecurity guidance (WHO/CDS/EPR/2006.6) of World Health Organization (WHO).

### DNA Extraction, Sequencing, and Analysis

Details of DNA extraction are shown in Supplementary Text S1. In brief, total DNA extraction of 36 soil samples was conducted (six groups with six repeats). DNA extraction of each sample possesses two technical replicates. The quality and concentration of extracted DNA were assessed. Samples were stored at −20°C before use. Details of bacteria 16S rRNA and fungi ITS gene amplicon sequencing are shown in Supplementary Text S2. Amplicon sequences were analyzed using the “DADA2” package in the R environment (version 3.6.1). Corresponding details are shown in Supplementary Text S3.

### Statistical Analysis

The bacterial community composition differences among treatments were tested by PERMANOVA (adonis, transformed data by Bray–Curtis, permutation = 999), implemented in R version 3.6.1. The DESeq function of the “DESeq2” package (version 1.18.1) was employed to test for differentially abundant ASVs among treatments. Statistical significance was based on a value of *p* < 0.05 (with FDR < 5% under the Benjamini–Hochberg correction).

## Results

### Correlation Between the Community Composition and Soil Properties

**FIGURE 1:**
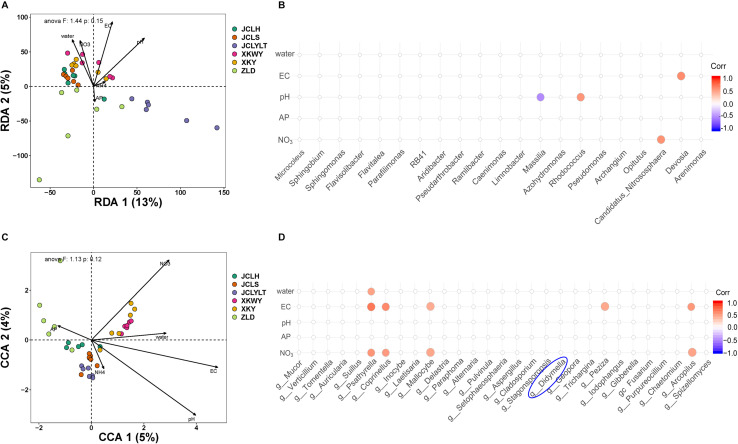
The influence degree of soil properties to the **(A)** bacterial community, **(C)** fungal community, and corresponding correlations between soil properties and **(B)** bacteria and **(D)** fungi.

The correlation between the community composition and soil properties was statistically analyzed based on six different types of soil. These parameters, including water-filled pore space (WFPS), pH, EC, AP, NO_3_^–^, and NH_4_^+^, are shown in Supplementary Table S1 and Figure 1. For the bacteria, the influence degree of the abovementioned parameters ranged as follows: pH > EC > WFPS > NO_3_^–^ > AP > NH_4_^+^ (Figure 1A). These soil properties contributed to 18% of the change in the bacterial community (Figure 1A). According to the results of the correlation analysis, the influence of pH mainly affected *Massilia* and *Rhodococcus*, EC had a higher influence on *Devosia*; and NO_3_^–^ on *Candidatus nitrososphaera*. For the fungi, the degree of influence of the abovementioned parameters ranged as follows: EC > pH > NO_3_^–^ > WFPS > AP > NH_4_^+^ (Figure 1C). These soil properties contributed to 9% of the change in the fungal community (Figure 1C). In addition, the influence of EC mainly focused on *Psathyrella*, *Coprinellus*, *Mallocybe*, *Peziza*, and *Arcopilus*; NO_3_^–^ mainly influenced *Psathyrella*, *Coprinellus*, *Mallocybe*, and *Arcopilus*; and WFPS mainly influenced *Psathyrella*.

### Morphological Characteristics and Identification of Endophytic Fungi

**FIGURE 2:**
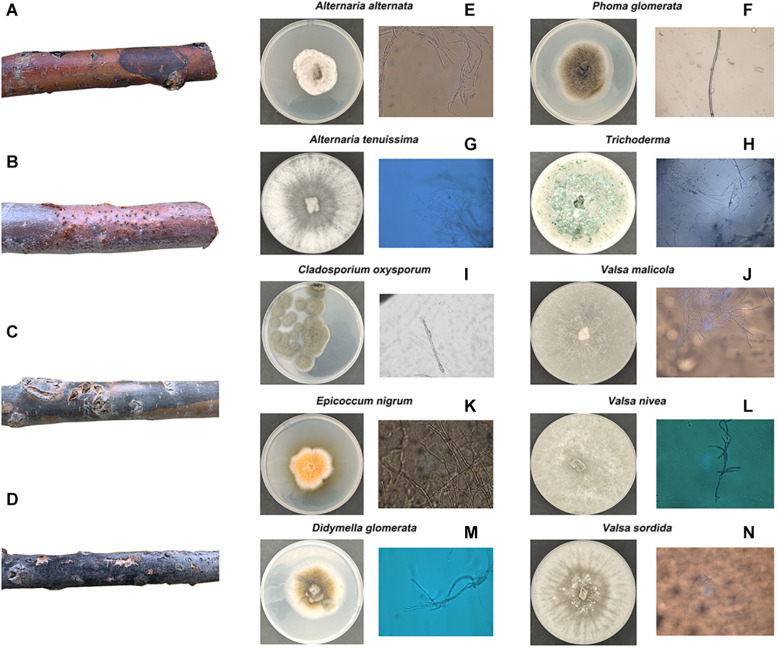
Four kinds of common morphological characteristics of diseased *Populus euphrasae* in XKY, ZLD, and JCL are shown in **(A–D)**. The endophytic fungi isolated and identified from **(A–D)** are shown in **(E–N)**.

To explore the correlations between stem pathogen infection and microbiome assemblage in the root soil, the endophytic fungi in the symptomatic segments were isolated and identified (Figure 2). A–D in Figure 2 is four typical symptoms of diseased *P. euphratica* in XKY, ZLD, and JCL. Finally, a total of 10 types of endophytic fungi were isolated and identified. Among these fungi, *Epicoccum nigrum* can produce some colored pigments, which are antifungal agents against other pathogenic fungi (Brown et al., 1987; Sun et al., 2011; Radić and Štrukelj, 2012), and *Alternaria* species (*Alternaria alternate*, *Alternaria tenuissima*), whose killing power release depends on a high-humidity environment (Moral et al., 2018). *Phoma glomerata*, which has an excellent ecological plasticity, can be permitted by 90 different kinds of plants and normally can cause leaf spot (Dörr et al., 2011). *Trichoderma* is a widespread fungus that is considered as an opportunistic avirulent plant symbiont (Harman et al., 2004; Bae et al., 2011). Previously, reports of *Cladosporium oxysporum* indicated that it has pathogenic effects on tomato and some vegetables, while it normally causes leaf spot (Wilingham et al., 2002; Baiswar et al., 2011; Zheng et al., 2014). *Didymella glomerata* is within the *Didymellaceae* family, which can cause stem lesions or cankers (Chen et al., 2015; Basim et al., 2016; Yao et al., 2016; Donati et al., 2018). The *Valsa* genus (*Valsasordida*, *Valsanivea*, *Valsamalicola*, and *Valsamali*) belongs to the family of Valsaceae, which can cause trunk diseases. Additionally, trunk diseases possess great power to kill young poplar trees 2 or 3 years after infection (Yi and Chi, 2011; Ma et al., 2016; Aghdam et al., 2017; Liu et al., 2018; Yu et al., 2018).

### Effects of *P. euphratica* on the Soil Microbial Community

**FIGURE 3:**
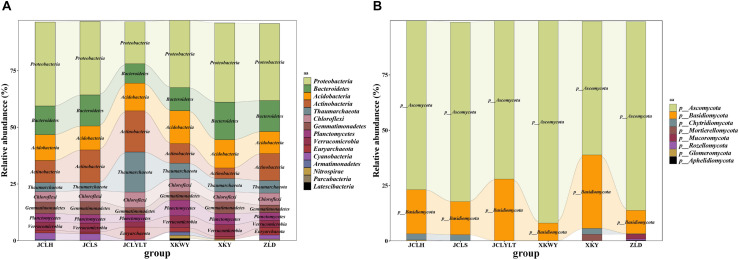
The relative abundance of **(A)** bacterial community and **(B)** fungal community.

According to the results of relative abundance analysis, for the bacteria, the most common phyla in the six types of soils were *Proteobacteria*, *Bacteroidetes*, *Acidobacteria*, *Actinobacteria*, and *Thaumarchaeota*. After *P. euphratica* planting, the relative abundance of *Proteobacteria* (dominant OTUs: OTU_12049, OTU_12644) (Supplementary Figure S1) and *Bacteroidetes* (dominant OTUs: OTU_13655, OTU_13821) (Supplementary Figure S1) increased (Figure 3A). However, the portions of *Actinobacteria* (dominant OTUs: OTU_22374) (Supplementary Figure S1) and *Thaumarchaeota* (dominant OTUs: OTU_5953, OTU_5954) (Supplementary Figure S1) were decreased (Figure 3A). At the same time, the fungi *Ascomycota* (dominant OTUs: OTU_3086, OTU_3548 and OTU_3550) (Figure 6) and *Basidiomycota* (dominant OTUs: OTU_2456) (Figure 6) were two of the most common fungal phyla. After *P. euphratica* planting, compared with the soil without *P. euphratica*, the relative abundance of *Ascomycota* in all the selected soils, except XKY, was increased (Figure 3B). However, the relative abundance of *Basidiomycota* was decreased in JCLH, JCLS, XKWY, and ZLD. Interestingly, XKY was the only exception (Figure 3B). By comparing the results of the relative abundance changing between the phyla level and the dominant OTUs level, we found that the relative abundance changing of similar trend dominant OTUs could meet with the phyla level well (Figures 3, 6 and Supplementary Figure S1).

#### α-Diversity

**FIGURE 4:**
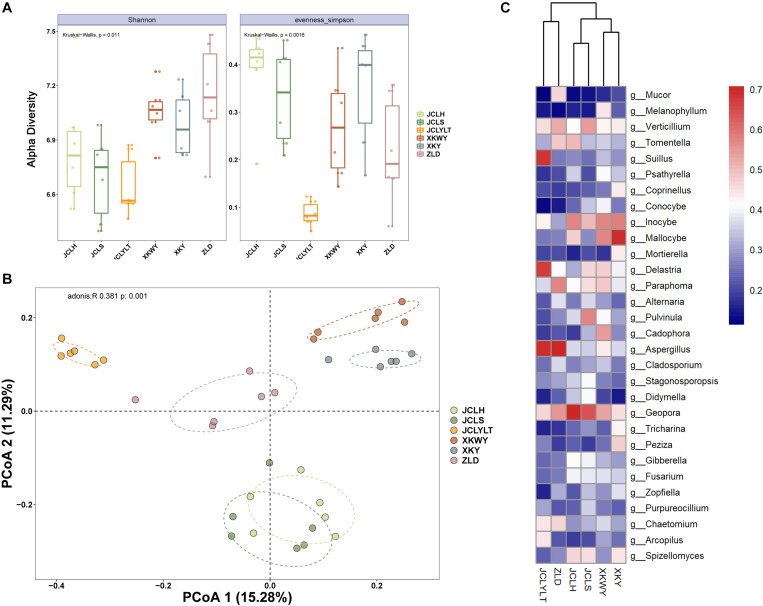
**(A)** Alpha diversity analysis based on the OTUs account for the species richness using eveness Simpson index and species diversity using Shannon index. **(B)** The Bray–Curtis dissimilarity principal coordinate analysis (PCoA) of bacterial community structures in different soils. **(C)** Characteristic bacterial populations with significant community differences in different soils.

**FIGURE 5:**
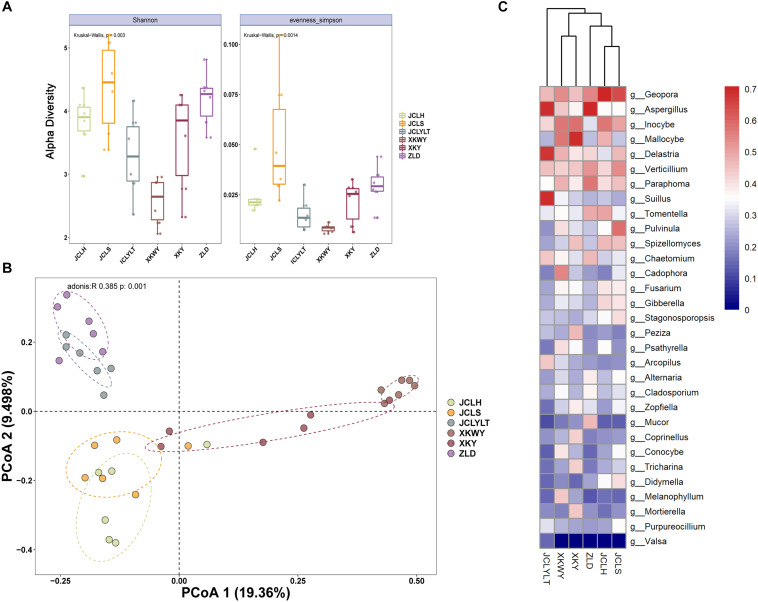
**(A)** Alpha diversity analysis based on the OTUs account for the species richness using eveness Simpson index and species diversity using Shannon index. **(B)** Bray–Curtis dissimilarity principal coordinate analysis (PCoA) of fungal community structures in different soils. **(C)** Characteristic fungal populations with significant community differences in different soils.

The results of the observed α-diversity are shown in Figures 4A, 5A, which indicated that *P. euphratica* planting could influence the community diversity of both bacteria and fungi significantly. Overall, the number of OTUs in the same soil sample was higher for bacteria than for fungi. For the bacteria, the community richness (Figure 4A, eveness Simpson) was increased after *P. euphratica* planting compared with JCLYLT. As for the species diversity (Figure 4A, Shannon), XKWY, XKY, and ZLD have higher species diversity than JCL. After *P. euphratica* planting, the species diversity difference of JCLH, JCLS, and JCLYL was not obvious. For the fungi, the community richness in JCLS was higher than that in other experimental sites (Figure 5A, eveness Simpson). Additionally, a similar tendency existed in the community diversity (Figure 5A, Shannon).

#### β-Diversity

For the bacterial community, the Bray–Curtis dissimilarity principal coordinate analysis (PCoA) results showed significant differences in the soils of JCL, XKWY, XKY, and ZLD (Figure 4B). In the JCL soils, after *P. euphratica* planting, both JCLS and JCLH showed pronounced differences compared to JCLYLT. The bacterial community between JCLS and JCLH was not completely separated. For the fungal community, the PCoA results indicated that the fungal community in the selected soil showed significant differences (Figure 5B). In the JCL soils, after *P. euphratica* planting, both JCLS and JCLH showed pronounced differences compared to JCLYLT. Similar to the bacterial community, the fungal community between JCLS and JCLH was not completely separated.

The differential abundance of bacteria and fungi of six selected soil sites are shown in Figures 4C, 5C. The phyla with significant community differences from all the selected OTUs were presented.

The differential abundances of bacteria and fungi of six selected soil sites are shown in Figures 4C, 5C. The phyla with significant community differences based on all the selected OTUs were presented.

## Discussion

The composition of soil microbiomes can be influenced by multiple environmental factors, and further interactions between pathogens and plants face the same situation (Xu et al., 2015; Wei et al., 2019). The results of this study indicated that soil physicochemical properties changed the composition of bacterial and fungal communities. Different parameters always possess, to some extent, a preference for specific phyla. For the bacteria, four different phyla were identified after taking the bacterial composition of all the sequenced soil samples into consideration. These affected phyla included *Massilia*, *Rhodococcus*, *Devosia*, and *Candidatus Nitrososphaera*. Previous studies have shown that most Rhodococcus species are benign (Mcleod et al., 2006). The genus *Massilia* possesses bacteria that are able to suppress pathogens for healthy plants. Studies of *Devosia* and *Candidatus Nitrososphaera* are scarce, and the pathogenicity of these genera remains unclear (Scola et al., 1998; Vannini et al., 2004; Zhalnina et al., 2014). For the fungi, EC, NO_3_^–^, and WFPS contributed some effect to five phyla, independent or synergistic. All the phyla had not been isolated and identified in symptomatic segments. Bacterial isolation and identification from infected segments were not studied here due to the large complexity.

Figures 4B, 5B show that the microbial community structure in the soils around the surviving *P. euphratica* is markedly different (*P* < 0.001). These results show the foundation difference of soil microbiome composition between different study sites. For the JCL sites, after *P. euphratica* planting, the microbiome community structures in both JCLH and JCLS were separated completely from JCLYLT (*P* < 0.001) (Figures 4B, 5B). At the same time, the soil bacterial and fungal community structures between JCLH and JCLS almost clustered into two distinct groups but were not thoroughly separated (*P* < 0.001, accounting for 26.57% of the bacterial community and 28.86% of the fungal community). Early reports showed that variations in soil microbiome community structures and functions can affect target plants and determine whether the plants survived or succumbed to disease (Wei et al., 2019). In addition to composition, microbiome diversity level is also an important predictor for the soil health and plant growth (Saleem et al., 2019). Therefore, significant soil microbiome community structures have a great potential to lead to the high death rate of *P. euphratica*.

**FIGURE 6:**
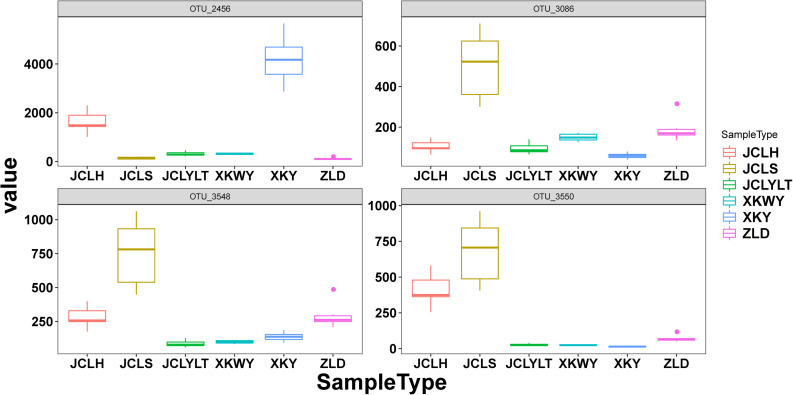
The relative abundance of OTUs 2456, OTUs 3086, OTUs 3548, and OTUs 3550 in the studied soils.

In this study, 10 types of endophytic fungi were isolated and identified. After systematic literature review, we found that *Alternaria* (*A. alternate*, *A. tenuissima*), *D. glomerata*, and the *Valsa* genus (*Valsa sordida*, *Valsa nivea*, *Valsa malicola*, *Valsa mali*) have great potential to participate in plant–pathogen interactions. Results showed that Ascomycota was the major fungal component in the selected soils with or without *P. euphratica* planting. Interestingly, *Alternaria*, *Didymella*, and *Valsa* all belong to the division of Ascomycota. By analyzing several characteristic fungal populations (the abundance of specific phyla in the studied soils that possess dramatic differences except *Valsa*, *p* < 0.05), we found that for the *Alternaria*, the relative abundance of *Alternaria* (OTU_3086, on behalf of *Alternaria alternate*) in JCLS was the highest within six selected soils (Figure 6A). The abundance of *Alternaria* in different studied sites followed an interesting rank: JCLS > ZLD > JCLH > XKWY ≈ XKY ≈ JCLYLT. The relative abundance of *Didymella* in JCLS was the highest. The abundance of *Didymella* (3,550 OTUs, on behalf of *D. glomerata*) in different studied sites followed an interesting rank: JCLS > JCLH > ZLD > XKWY ≈ XKY ≈ JCLYLT (Figure 6B). For the genus *Valsa*, there were almost no differences in the relative abundance among the selected soils. In this respect, the high death rate of *Populus* may be caused by *Alternaria alternate* and *D. glomerata*.

According to the plant–pathogen interactions discovered in the fungi perspective, we found that higher relative abundance of the target fungi in the *P. euphratica-*planted soil poses a high risk of infection. When we do a reasonable extrapolation of this result in the bacteria view, several candidate bacteria (OTU_12049 and OTU_12644, on behalf of JG34-KF-161 and *Sphingomonadaceae Sphingomonas*, respectively, within the *Proteobacteria* phyla) could be listed (Supplementary Figure S1). In addition, we should also pay attention to the relative abundance decrease of *Actinobacteria* (dominant OTUs: OTU_22374) (Supplementary Figure S1) and *Thaumarchaeota* (dominant OTUs: OTU_5953, OTU_5954) (Supplementary Figure S1) after *P. euphratica* planting. *Thaumarchaeota* has been regarded as the key player within the global nitrogen cycle (Shao et al., 2019). The phylogeny and the function of the class Actinobacteria remains controversial up to now (Ludwig et al., 2012; Sen et al., 2014). Its function within the plant–pathogen interaction system should be explored thoroughly. So, to do a further confirmation about this prediction will be a challenging, but meaningful, object in our future studies.

## Conclusion

In conclusion, the results of the present study revealed deeper interactions between plant and pathogen. Increased attention on the presence of pathogens during tree planting will be a crucial factor in plant survival rates. Massart and Mark point out that in order to achieve a better biological control, we must combine as much potential factors together (e.g., host plants, host genotype, integrate microbial community, pathogen, biocontrol agents, molecules, etc.) (Massart et al., 2015; Mazzola and Gu, 2002). In addition, previous studies have indicated that the tolerance of plants to pathogens increases with increasing tree age and growth state (Mansfeld et al., 2017; Berendsen et al., 2018). Plant–pathogen interactions as a complicated dynamic equilibrium process warrant further study.

## Data Availability Statement

The data can be found in the NCBI database, with accession number – PRJNA597436.

## Author Contributions

YT puts forward the whole idea of this manuscript and implemented all the tasks. ZD had given several constructive instructions to this manuscript. XW and FT had given site instructions and integtate theory with practice. BG helped ZD with some interdisciplinary theories. CZ works as a assistant and made many contributions to this manuscript. All authors contributed to the article and approved the submitted version.

## Conflict of Interest

The authors declare that the research was conducted in the absence of any commercial or financial relationships that could be construed as a potential conflict of interest.
