# Supplementary material for: Metagenomics Reveal Correlations Between Microbial Organisms in Soils and the Health of Populus euphratica
Source: Front Microbiol. 2020 Sep 8;11:2095. doi: 10.3389/fmicb.2020.02095 (PMC7506035; doi:10.3389/fmicb.2020.02095)
Supplement: Supplementary file 1 [file Data_Sheet_1.docx]

**Metagenomics reveal correlations between** **microbial organisms in soils and the health of *Populus euphratica***

Yu Tuo^1^*,* ZhiBao Dong^1^[[1]](#footnote-1)^*^*,* XiPing Wang^2^*,* BeiBei Gao^3^*,* ChunMing Zhu^1^ *,* Fei Tuo^4^

1. School of Geography and Tourism, Shaanxi Normal University, Xi'an, Shannxi Province 710062, China.
2. College of Horticulture, Northwest Agriculture and Forestry University, no.3 tai cheng road, YangLing demonstration zone, Shaanxi province.
3. Department of Pesticide Science, College of Plant Protection, Nanjing Agricultural University, State and Local Joint Engineering Research Center of Green Pesticide Invention and Application, Nanjing, Jiangsu 210095, China
4. Greening committee office of Forestry Bureau of Yulin city, Shahe road Yuyang district, Yulin, Shaanxi, 719000, China.

*** Correspondence:**

Corresponding author: ZhiBao Dong.

Email address: zbdong@snnu.edu.cn (Z. Dong)

**Text S1 DNA extraction**

Total DNA was extracted from a total of 36 soil samples (6 for each treatment) with 0.5g soil using Power Lyzer PowerSoil DNA Isolation Kit (Qiagen, Germany) following the manufacturer's protocol. Measurement of DNA quality and quantity was performed with 1% agarose gel and NanoDrop 2000 spectrophotometer (Thermo Scientific, Waltham, MA, USA). DNA was extracted from two technical replicates per sample to minimize the DNA extraction bias. Samples were stored at -80 °C, and technical replicates were pooled before performing polymerase chain reaction. The DNA quality was assessed according to the 260/280-nm and 260/230-nm absorbance ratios using a NanoDrop ND-2000 spectrophotometer (NanoDrop, ND2000, Thermo Scientific, 111 Wilmington, DE). The concentration of the extracted DNA was between 40 and 60 ng/μL.

**Text S2 PCR amplification, and Illumina sequencing**

Bacterial 16S rRNA gene fragments were amplified from the extracted DNA using primers 515F, GTGYCAGCMGCCGCGGTAA, and 806R, GGACTACNVGGGTWTCTAAT) (Walters et al. 2016), and the ITS2 region were performed to profiling fungal communities with the primers ITS3-F: GCATCGATGAAGAACGCAGC; ITS4-R: TCCTCCGCTTATTGATATGC).^1^ The following PCR conditions: 30 s at 95 °C, 30 s at 59 °C, and 30 s at 72 °C for 30 cycles. PCRs were performed in a total volume of 25 μL with 9.75 μL of water, 5 μL of 5× PCR buffer, 5 μL of 5× Q5 GC high enhancer, 2 μL of deoxyribonucleotide triphosphates (dNTPs), 1 μL of each primer, 0.25 μL of 5 U/μL Q5 polymerase, and 1 μL of extracted DNA. After PCR amplification, Products were run on a 1% agarose gel and those with a clear band between 290-310 bp were combined for sequencing. PCR products were mixed in equidensity ratios according to the GeneTools Analysis Software (Version 4.03.05.0, SynGene). Then, mixture of PCR products was purified with EZNA Gel Extraction Kit (Omega, USA). Amplicons were subjected to paired-end sequencing on the Illumina MiSeq sequencing platform (Illumina Inc., USA) using PE250 at the Genomics Core of Michigan State University. After assembly, we obtained ~ 290 bp reads covering the V4 region of the bacterial 16S rRNA.

**Text S3 Amplicon sequence processing and analysis**

Before sequences analysis, quality filtering on the paired-end raw reads was performed under specific filtering conditions to obtain the high-quality clean reads according to the Trimmomatic (V0.33, http://www.usadellab.org/cms/?page=trimmomatic). Then, sequences were analyzed using the "DADA2" package in the R environment for sequence filtering, assembly and pick actual sequence variants. Sequences were truncated at base 120 and trimmed until base 15. This resulted in relatively short reads (~ 120 bp) of high quality from which actual sequence variants (ASVs) were identified.

The DADA2 pipeline then produced an ASV count table containing 0.24 million usable reads and ~ 8000 ASVs. Taxonomic assignment of ASVs was performed using the assignTaxonomy function in R environment and the SILVA (ver.123) 16S rRNA database. Statistical analyses of the 16S rRNA microbiome sequencing data were performed using the phyloseq package in the R environment (R, 2019, https://www.r-projec-t.org version 3.6.1). Before calculation of *beta* diversity, the relative abundance was used to standardize the OTU profiles and Bray-Curtis similarity matrices were prepared using the beta_diversity.py script (Qiime1.9.0). PERMANOVA (Adonis, transformed data by Bray-Curtis, permutation = 999) was used to determine if *beta* diversity differed from treatments. The Principal coordinate analysis (PCoA) plots were generated from Bray-Curtis similarity matrices created using R package ggplot2package (version 2.2.1).

**Table S1 Characteristics of different soils**

| Study site | NH_4_^+^ (mg/kg) | NO_3_^-^  (mg/kg) | AP  (mg/kg) | pH  (1:5 H_2_O) | EC  (1:5 H_2_O, ms m^-1^) | WFPS |
| --- | --- | --- | --- | --- | --- | --- |
| ZLD | 14.37 | 4.42 | 12.12 | 8.7 | 37.50 | 0.064 |
| JCLH | 17.60 | 5.74 | 12.41 | 9.5 | 55.83 | 0.063 |
| JCLS | 14.67 | 5.24 | 12.35 | 9.5 | 55.66 | 0.058 |
| XKY | 16.92 | 12.55 | 12.81 | 9.5 | 67.08 | 0.138 |
| XKWY | 12.51 | 10.19 | 12.44 | 9.3 | 66.82 | 0.055 |
| JCLYL | 12.81 | 4.22 | 12.5 | 7.9 | 18.39 | 0.016 |


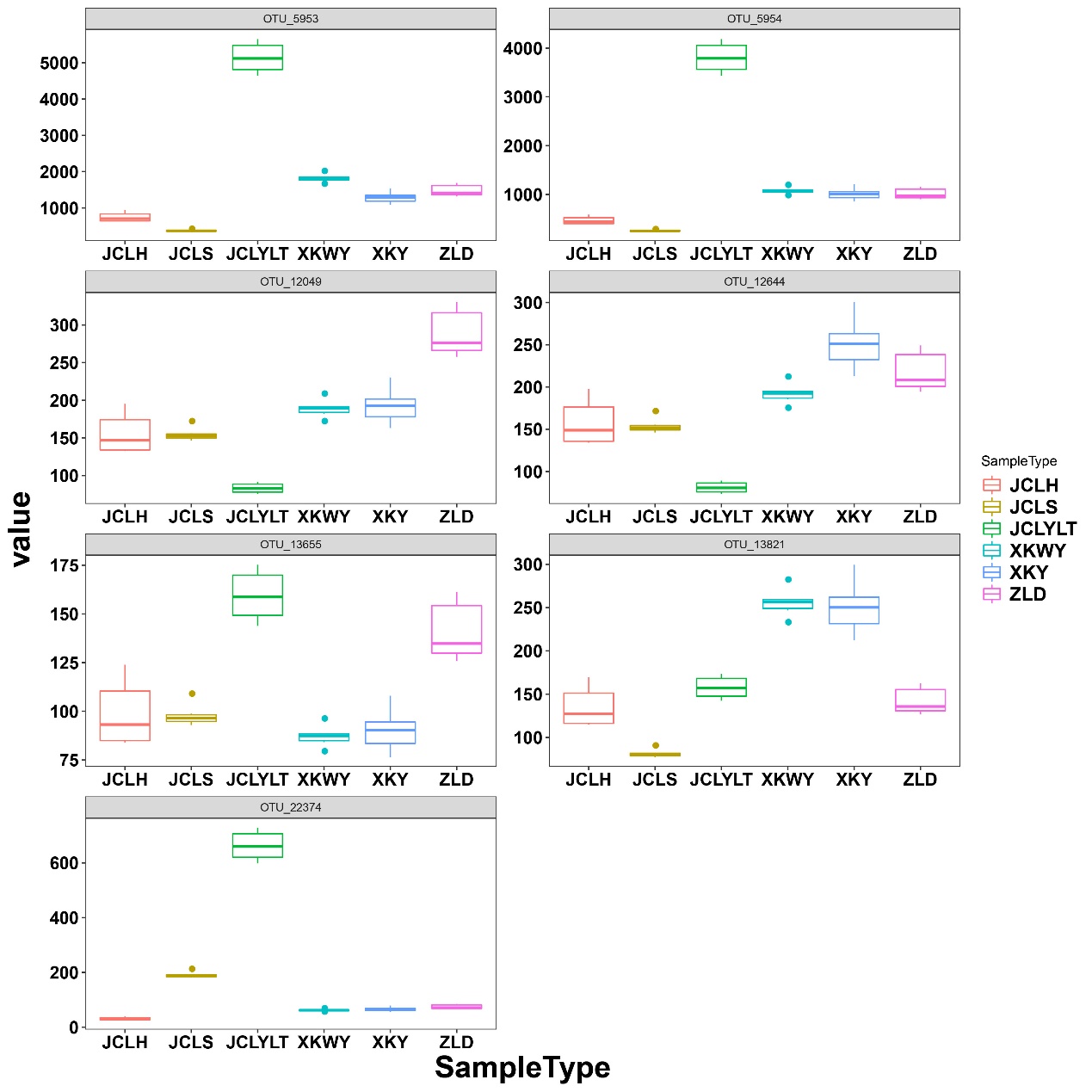


Figure S1. The relative abundance of OTUs 5953, OTUs 5954, OTUs 12049, OTUs 12644, OTUs 13655, OTUs 13824, and OTUs 22374 in the studied soils

**Reference**

1. McKay, G., Brown, A., Bjourson, A., & Mercer, P. Molecular characterisation of Alternaria linicola and its detection in linseed. Eur. J. Plant Pathol. **105**: 157-166. (1999).

1. [↑](#footnote-ref-1)
